# Management of drug-induced liver injury associated with anti-cancer therapy

**DOI:** 10.3389/fphys.2025.1541020

**Published:** 2025-03-27

**Authors:** Bruno Vincenzi, Mao Yimin, Raúl J. Andrade, Mauricio Morales Castillo, Gamar Akhundova-Unadkat, José M. Mato

**Affiliations:** ^1^ Medical Oncology, Policlinico Universitario Campus Bio-Medico, Rome, Italy; ^2^ Division of Gastroenterology and Hepatology, Renji Hospital, Shanghai Jiao Tong University School of Medicine, NHC Key Laboratory of Digestive Diseases, Shanghai Research Center of Fatty Liver Disease, Shanghai, China; ^3^ Servicios de Aparato Digestivo, Instituto de Investigación Biomédica de Málaga IBIMA_Plataforma Bionand, Hospital Universitario Virgen de la Victoria, Universidad de Málaga, Málaga, Spain; ^4^ CIBERehd, Madrid, Spain; ^5^ Abbott Products Operations AG, Allschwil, Switzerland; ^6^ CIC bioGUNE, CIBERehd, Parque Tecnológico de Bizkaia, Derio, Spain Parque Tecnológico de Bizkaia, Derio, Spain

**Keywords:** anti-cancer therapy, drug-induced liver injury, hepatoprotective drugs, liver enzyme abnormalities, narrative review

## Abstract

Drug-induced liver injury (DILI) is a leading cause of drug withdrawal, a particular cause for concern among patients receiving anti-cancer treatment. This review summarizes the available evidence on the efficacy of hepatoprotective drugs in normalizing liver enzyme abnormalities among patients with DILI due to treatment with anti-cancer therapies. Across relevant publications, the effects of several compounds on anti-cancer therapy-induced DILI were assessed. Treatment with hepatoprotective agents which is usually initiated after DILI has been detected and involves cessation of causative anti-cancer therapy, has demonstrated improvements in liver enzyme elevation. However, prophylactic treatment with two agents in particular, ademetionine and bicyclol have shown hepatoprotective effects that enabled patients to continue with their anti-cancer therapy with a reduced subsequently reduced risk of hepatotoxicity. While these publications show some evidence for the benefits of hepatoprotective agents among patients with DILI due to anti-cancer therapy, more research is needed to fully determine the effects of hepatoprotective drugs in resolving DILI signs and symptoms among patients receiving treatment for cancer.

## Introduction

The liver is a prime target for medication-induced damage due to its central role in drug metabolism (Yuan and Kaplowitz, 2013). Drug-induced liver injury (DILI) may be caused by a broad range of pharmaceutical agents including clinical medications and herbal and dietary supplements (Chalasani et al., 2021; Pinazo-Bandera et al., 2023). It is the most common cause of acute liver failure (ALF), and consequently, liver transplant, in both Europe and the US (Bernal and Wendon, 2013; Lee, 2003; Katarey and Verma, 2016). Furthermore, it is a leading cause of drug withdrawal (Ye et al., 2018). In an analysis of 133 drugs withdrawn from the market due to safety reasons between 1990 and 2010, the most common reasons were hepatotoxicity (27.1%) and cardiotoxicity (18.8%) (Craveiro et al., 2020). The true incidence of DILI varies from country to country and across populations and is challenging to estimate with the true incidence likely to be higher than reported (Li et al., 2022a). In population studies, the annual incidence is reported between 2.7 and 19 per 100,000 persons per year (Björnsson, 2024). A recent systematic review and meta-analysis of 14 population-based studies reported the overall incidence of DILI as 4.94 per 100,000 person-years (95% CI: 4.05–5.83), with the highest incidence in Asia (17.82 per 100 00) person-years [95% CI: 6.26–29.38] (Li et al., 2023).

DILI may be intrinsic (or direct) or idiosyncratic. Intrinsic DILI is usually predictable and dose-related with a relatively short time to onset, while idiosyncratic DILI occurs less frequently and is usually unrelated to dose (Brennan et al., 2022). One of the major causes of idiosyncratic DILI is anti-cancer therapy. Anti-cancer therapies known to increase the risk of DILI include chemotherapy, tyrosine kinase inhibitors, and immunotherapies such as immune checkpoint inhibitors that target cytotoxic T-lymphocyte-associated antigen 4 (CTLA-4) and programmed cell death protein (PD-1) (Vincenzi et al., 2018). DILI caused by anti-cancer therapy is unpredictable, unrelated to dose, and is the leading cause of dose reductions or cycle delays. Furthermore, it can affect outcomes and subsequent treatment choices (Mudd and Guddati, 2021; Vincenzi et al., 2016; Cunningham et al., 2024). The mainstay of treatment for DILI is early recognition and withdrawal of the likely causative agent(s), assessment of liver injury, and close observation for resolution (Ye et al., 2018; Brennan et al., 2022). Chemotherapy agents are hepatotoxic through multiple pathways, thus producing different types of liver injury including elevation of liver enzymes, drug-induced hepatitis, veno-occlusive disease, steatohepatitis, fibrosis and liver failure (Vincenzi et al., 2018; Mudd and Guddati, 2021; Vincenzi et al., 2016). For example, oxaliplatin may cause elevations of liver enzymes but is commonly associated with sinusoidal and vascular injury to the liver which can lead to sinusoidal obstruction syndrome (Vincenzi et al., 2018; Mudd and Guddati, 2021). Immune checkpoint inhibitor-associated hepatoxicity generally manifests as autoimmune hepatitis, although immune-mediated cholangitis can also occur (Vincenzi et al., 2018; Cunningham et al., 2024). In some cases, spontaneous recovery from hepatotoxic effects occurs, without the need for supportive measures (Cunningham et al., 2024; Dara and Ghabril, 2024; Da et al., 2022). Therapeutic options for DILI are very limited. Corticosteroids may be useful in instances where acute autoimmune hepatitis cannot be excluded or to treat hepatotoxicity due to immune checkpoint inhibitors (Brennan et al., 2022; Andrade et al., 2019). Other immunosuppressants such as mycophenolate mofetil have also been used either alongside or as an alternative to corticosteroids (Andrade et al., 2019). Hepatoprotective drugs that have been used for DILI aim to improve liver function, promote liver cell regeneration and/or enhance liver detoxification (Niu et al., 2021). Although there is no unified classification, they can be grouped by mechanism of action into detoxification drugs (e.g., N-acetylcysteine and glutathione), anti-inflammatory drugs (e.g., glycyrrhizic acid), hepatocyte membrane protectors (e.g., polyene phosphatidylcholine), and antioxidants (e.g., bicyclol, silymarin) (Li et al., 2021).

Ademetionine (AdoMet), the main product of methionine metabolism, has been proposed as a therapeutic option for cholestasis owing to DILI. Ademetionine plays a key role in methylation reactions, epigenetic regulation, detoxification reactions, phospholipid synthesis, and glutathione synthesis. It is indicated (via oral, intravenous, or intramuscular routes) for the treatment of adults with intrahepatic cholestasis (IHC) in pre-cirrhotic and cirrhotic stages, as well as IHC in pregnancy, depressive symptoms, and relief of fatigue caused by chronic liver disease; studies have shown anti-proliferative effects in models of breast, colorectal, and liver cancers (Li et al., 2015; Ansorena et al., 2002; Lu et al., 2009; Lu and Mato, 2008). This review focuses on available evidence for the efficacy of hepatoprotective drugs in preventing liver enzyme abnormalities among patients with DILI due to treatment with anti-cancer therapies.

## Studies assessing hepatoprotective agents in DILI caused by anti-cancer therapies

Several studies have assessed the effects of hepatoprotective agents among patients with DILI due to anti-cancer therapies (Table 1). These therapies include ademetionine, bicyclol, glucocorticoids, magnesium isoglycyrrhizinate, monoclonal antibodies and silymarin.

**TABLE 1:** Summary of study designs, patient characteristics, DILI cause and type and clinical outcomes for the identified studies.

Authors	Study design	N =	Drug causing DILI	Type of liver damage	Drug administered for DILI treatment or prevention (including dose/duration)	Clinical outcome
*Ademetionine*						
Santini et al. (2003)	Observational, prospective	50	Three regimens Raltitrexed and oxaliplatin Irinotecan, 5-fluorouracil and folinic acid (FOLFIRI) Cyclophosphamide, methotrexate and 5-fluorouracil (CMF)	Elevation of serum transaminases and cholestasis markers	Ademetionine 400 mg twice a day in the intervals between chemotherapy cycles, initiated at first recognition of liver toxicity	ALT, AST and LDH were significantly reduced after 1 week, and further confirmed after 2 weeks. Beneficial effects persisted in the following chemotherapy courses
Vincenzi et al. (2011)	Observational, retrospective	105	FOLFOX IV (oxaliplatin, leucovorin, 5-FU)	Various including steatosis, steatohepatitis and sinusoidal dilatations	Ademetionine 400 mg BD prevention starting from beginning to end of chemotherapy (n = 40)	AST, ALT, GGT, and bilirubin were significantly lower in ademetionine patients. Also experienced lower grade of liver toxicity, reduced need for course delay and dose reduction
Vincenzi et al. (2012)	Observational, retrospective	78	Bevacizumab and XELOX (oxaliplatin, capecitabine)	Various including steatosis, steatohepatitis and sinusoidal dilatations	Ademetionine 400 mg BD prevention starting from beginning to end of chemotherapy (n = 32)	AST, ALT, LDH, GGT, and bilirubin were significantly lower in ademetionine patients. Also experienced lower grade of liver toxicity, reduced need for course delay and dose reduction
*Bicyclol*						
Li et al. (2014)	Randomized, prospective, controlled	300	Various: most common were a combination of oxaliplatin and fluoropyrimidine, oxaliplatin + capecitabine, FOLFOX	Elevation of transaminases and total bilirubin	Bicyclol (4,40-dimethoxy-5,6,50,60-dimethylene-dioxy-2- hydroxymethyl-20-carbonylbiphenyl) 25 mg orally three times a day on initiation of chemotherapy until end of all cycles (n = 147)	Incidence of grade I–IV elevation of serum transaminase and/or bilirubin was significantly lower in the prophylactic group (17.1%) vs. control (47.1%). Incidence of grade II–IV hepatic injury was also significantly lower in the prophylactic group (0.7%) vs. control group (12.4%)
*Corticosteroids*						
De Martin et al. (2018)		16/536 developed hepatitis	9 anti-PD-1/PD-L1 and 7 anti-CTLA-4 mAbs (monotherapy or in combination with anti-PD-1)	Hepatitis	7 oral corticosteroids at 0.5–1 mg/kg/day; 2 maintained on 0.2 mg/kg/day corticosteroids; and 1 required pulses and 2.5 mg/kg/day of corticosteroids, and the addition of a second immunosuppressive drug	Management tailored according to the severity of both the biology and histology of liver injury and while 7 improved spontaneously the remainder required steroids
Gauci et al. (2018)	Retrospective analysis (letter)	10/128 developed hepatitis	6 anti-CTLA4, 3 anti-PD-1 and 1 combination of anti-CTLA4 and anti-PD-1	Immune-related hepatitis	5 corticosteroids	Hepatitis resolved in all 5 patients The other 5 patients, as per hepatologist’s recommendation, did not receive steroids. Resolution observed in all cases
Miller et al. (2020)	Not stated	100/5,762 developed hepatotoxicity	Immune checkpoint inhibitors	ALT >5x ULN	Steroids (67 patients)	10/67 (14%) who received steroids had recurrent hepatotoxicity after the steroid taper. 31 patients resumed ICIs after ALT improvement, 8 (26%) developed recurrent hepatotoxicity. Characteristics of liver injury, response to steroids, and outcomes were similar between 38 individuals with and 62 without possible pre-existing liver disease
Swanson et al. (2022)	Retrospective analysis	112 (6 with confirmed DILI)	Durvalumab (n = 4), pembrolizumab (n = 1), paclitaxel (n = 1)	DILI	3 patients required corticosteroids; durvalumab was discontinued in 5 patients	All DILI cases had normalization of their liver biochemistries within a median of 52 days of follow up
*Magnesium isogycyrrhizinate*						
Tang et al. (2012)	Multicenter, randomized, double-blind phase 3 study	55	Methotrexate (n = 48) Epirubicin (n = 5) Gemcitabine (n = 2)	Chemotherapy-induced acute liver dysfunction	Randomly divided 2:1 to receive magnesium isoglycyrrhizinate (n = 35; 200 mg/d) or tiopronin (n = 20; 200 mg/d) for 2 weeks	Overall response in magnesium isoglycyrrhizinate group after 1 week was much higher (91.4%) than the tiopronin group (65%, p < 0.05). After 2 weeks, normalization of ALT and AST was significantly higher in magnesium isoglycyrrhizinate group than in the tiopronin-treated (42.1% and 37.5%) (p < 0.05)
*Monoclonal antibodies*						
Rubbia-Brandt et al. (2010)	Multi-institutional case series of surgically resected colorectal liver metastases	274	Oxaliplatin	Sinusoidal obstruction syndrome (histological evaluation)	Oxaliplatin/bevacizumab (n = 70) compared with the group treated by oxaliplatin alone (n = 204)	111 patients treated by surgery alone had no lesions. Hepatic lesions were less severe in patients treated with bevacizumab (n = 70) compared with those treated with oxaliplatin alone (n = 204): moderate/severe sinusoidal obstruction syndrome (31.4% vs. 62.2%); peliosis (4.3% vs. 14.6%); nodular regenerative hyperplasia (11.4% vs. 28.9%); centrilobular/venular fibrosis (31.4% vs. 52%); all p < 0.001)
*Silymarin (flavonoid extract from Silybum marianum)*						
Moezian et al. (2022)	Randomized, triple-blind, placebo-control	30	Doxorubicin, cyclophosphamide, paclitaxel	Chemotherapy-induced hepatotoxicity	Silymarin 140 mg three times daily (n = 15)	No difference in LFTs. Silymarin group had a nonsignificant trend towards less severe hepatic involvement
*Ursodeoxycholic acid*						
Saif et al. (2012) and Robles-Díaz et al. (2021)	Prospective, randomized, parallel control pilot	39	Chemotherapy	Induce apoptosis in hepatocytes and causes oxidative tissue	UDCA (10–15 mg/kg/day) with chemotherapy for 6 months, then followed up for 3 months after UDCA discontinued (n = 19) or received chemotherapy without UDCA and followed up for 9 months (n = 20)	Decreased levels of hepatic transaminases when UDCA concomitantly administered with chemotherapy. New increase in ALT after stopping UDCA during follow up period in the UDCA group
Bordbar et al. (2018)	Open label, randomized	80	Methotrexate	Induce apoptosis in hepatocytes and causes oxidative tissue	Group 1 oral vitamin E 400 mg/day (n = 20); group 2 oral UDCA 15 mg/kg/day (n = 19); group 3 UDCA and vitamin E (n = 19); and group 4 control group (n = 20)	Group 1: a slight increase in total bilirubin levels compared to baseline (P = 0.036). Group 2: a non-significant decline in AST and ALT levels during the study and 6 months after drug discontinuation. No evidence of significant fibrosis on liver fibroscan for any patients

5-FU, 5-fluorouracil; ALP, alkaline phosphatase; ALT, alanine transaminase; AST, aspartate aminotransferase; BD, twice daily; CMF, cyclophosphamide, methotrexate and 5-fluorouracil; DILI, drug-induced liver injury; FOLFIRI, irinotecan, 5-fluorouracil and folinic acid; FOLFOX, oxaliplatin, leucovorin, 5-fluorouracil; IV, intravenous; ICI, immune checkpoint inhibitor; LDH, lactate dehydrogenase; LFT, liver function test; mAB, monoclonal antibody; TBIL, total bilirubin; UDCA, ursodeoxycholic acid; ULN, upper limit of normal.

## Ademetionine

Across 3 studies, 1 prospective study (Santini et al., 2003) and 2 retrospective analyses (Vincenzi et al., 2012; Vincenzi et al., 2011), ademetionine improved or prevented liver enzyme abnormalities among patients with DILI. In the prospective study, AST, ALT and LDH levels were significantly reduced after 1 week of therapy (Table 1) and the effects on these enzymes persisted in subsequent chemotherapy courses allowing patients to receive their scheduled chemotherapy courses with a minimal number of dose reductions or administration delays (Santini et al., 2003). In both retrospective analyses, ademetionine was administered from the beginning to the end of chemotherapy as a preventative measure. Treatment with (compared to without) ademetionine was associated with significantly lower median levels of AST, ALT and γ-GT at the end of adjuvant therapy an also a lower grade of liver toxicity and a reduced need of course delay and dose reduction (Table 1) (Vincenzi et al., 2012; Vincenzi et al., 2011).

## Bicyclol

Bicyclol, a synthetic drug developed in China, is used to treat inflammatory liver injury. It protects liver cells by stabilizing cell membranes, scavenging free radicals, modulating oxidative stress, inhibiting inflammatory cytokines and promoting autophagic flux (Benić et al., 2022; Zhao et al., 2020). The effect of bicyclol on anti-cancer therapy-induced DILI was assessed in a 2014 study (Zhao et al., 2020). Among 300 patients aged ≥60 years treated for cancer (included colorectal, lung, gastric, lymphoma, pancreas, bile duct and ampulla), incidence of grade I-IV elevation of serum transaminase and/or bilirubin was significantly lower in patients treated with chemotherapy supplemented with daily bicyclol compared with patients treated with chemotherapy alone (Table 1). Chemotherapy included combination regimens with oxaliplatin and fluoropyrimidine, docetaxel, gemcitabine, irinotecan, etoposide and paclitaxel. There were no significant differences between groups regarding cancer types, disease staging, previous chemotherapy and complicating liver metastasis. The incidence of grade II-IV hepatic injury was also significantly lower in the prophylactic group than in the control group (Li et al., 2014). In addition, results of a multicenter, randomized, phase II trial demonstrated the efficacy of bicyclol (low-dose 25 mg times a day, high-dose 50 mg TID vs. polyene phosphatidylcholine control) in the treatment of patients with idiosyncratic acute DILI with significant higher ALT normalization rates at weeks 1, 2, 4, 6 and 8 compared with polyene phosphatidylcholine control. Bicyclol has been included in local guidelines based on this data and a phase 3 trial is ongoing (Tang et al., 2022). It is also useful to note that Abbott sold its non-U.S. developed markets specialty and branded generics business to the company Viatris on 27 February 2015.

## Corticosteroids

Glucocorticoids are often used in the management of DILI owing to their anti-inflammatory, immunosuppressive and antiallergic effects (Li et al., 2022b). Across four retrospective studies among patients treated with corticosteroids for DILI (De Martin et al., 2018; Gauci et al., 2018; Miller et al., 2020; Swanson et al., 2022), most DILI cases resolved following corticosteroid treatment, with a small number requiring maintenance low-dose corticosteroids or additional treatment (Table 1). The first retrospective analysis included 536 patients treated with anti-PD-1/PD-L1 or CTLA-4 immunotherapies, 16 of whom developed grade ≥3 hepatitis and underwent liver investigations. These included viral assays, autoimmune tests and liver biopsy, histological review, and immunostaining of liver specimens (De Martin et al., 2018). Treatment was tailored depending on the severity of liver injury; six patients improved spontaneously, seven received oral corticosteroids at 0.5–1 mg/kg/day, two were maintained on 0.2 mg/kg/day corticosteroids, and one required 2.5 mg/kg/day corticosteroids and the addition of a second immunosuppressive drug. It is important to note that in 37.5% (6/16) patients treated with immune checkpoint inhibitors, the biochemical abnormalities of DILI improved spontaneously, and thus did not require any treatment (De Martin et al., 2018). In the second retrospective analysis, among 10 patients with hepatitis due to anti-CTLA-4 and/or anti-PD-1, hepatitis resolved in 5 patients following corticosteroid treatment and spontaneously in the other 5 patients (Gauci et al., 2018). A total of 100 of 5,762 patients developed DILI after receiving immune checkpoint inhibitor treatment in the third retrospective analysis (Miller et al., 2020). Of the 67 patients who received steroids, only 10 had recurrent hepatotoxicity after steroids taper. Finally, in a retrospective analysis of 6 patients with confirmed DILI (4 following durvalumab treatment, 1 following pembrolizumab treatment and 1 following paclitaxel treatment), 3 patients required corticosteroids, and all DILI cases resolved within a median of 52 days of follow up (Swanson et al., 2022).

## Magnesium isoglycyrrhizinate

Magnesium isoglycyrrhizinate, refined from glycyrrhizic acid which is extracted from the roots of herb *Glycyrrhiza glabra*, has been shown to have anti-inflammatory, anti-oxidative and hepatoprotective effects (Benić et al., 2022; Li et al., 2022b). It scavenges free radicals, prevents the increase of serum transaminase, reduces hepatocyte degeneration, and reduces necrosis and inflammatory cell infiltration (Benić et al., 2022; Tang et al., 2015). In a multicentre, randomized, double-blind study comparing the effects of magnesium isoglycyrrhizinate with tiopronin among 55 patients with chemotherapy-induced DILI due to methotrexate, epirubicin and gemcitabine (Tang et al., 2012), overall response was significantly higher among patients receiving magnesium isoglycyrrhizinate than patients receiving troponin (Table 1). Furthermore, significantly higher proportions of patients on magnesium isoglycyrrhizinate achieved ALT and AST normalisation after 2 weeks compared with those on tiopronin (Table 1).

## Monoclonal antibodies

The effects of the monoclonal antibody bevacizumab on DILI were assessed among patients with colorectal liver metastases treated with oxaliplatin (Rubbia-Brandt et al., 2010). Hepatic lesions identified through histological evaluation were less severe in patients treated with oxaliplatin/bevacizumab compared with those treated with oxaliplatin alone, with a lower incidence of moderate/severe sinusoidal obstruction syndrome (31.4% versus 62.2%), peliosis (4.3% versus 14.6%), nodular regenerative hyperplasia (11.4% versus 28.9%) and centrilobular/venular fibrosis (31.4% versus 52%). However, the hepatoprotective effects of bevacizumab were not evaluated further.

## Silymarin

Silymarin, a flavonoid extracted from the seeds and fruit of *Silybum marianum*, with antioxidant, antifibrotic and anti-inflammatory properties, has been used as a hepatoprotective agent (Gillessen et al., 2022; Tao et al., 2019). A randomized, triple-blind, placebo-controlled trial in 30 patients with non-metastatic breast cancer assessed the effects of silymarin on DILI induced by treatment with doxorubicin/cyclophosphamide-paclitaxel (AC-T) (Moezian et al., 2022). There was a non-significant trend towards more severe liver involvement in patients receiving placebo compared with those receiving silymarin, based on ultrasonography (p = 0.083). However, no between-group differences in liver involvement were seen based on FibroScan and liver function tests.

## Ursodeoxycholic acid

Ursodeoxycholic acid (UDCA) has an anti-apoptotic effect on hepatocytes by stabilizing the cell membrane and can protect from damage induced by anti-cancer therapies (Goossens and Bailly, 2019; Bordbar et al., 2018). For example, in a prospective randomized parallel study in 39 children with acute lymphoblastic leukemia (ALL) randomized to receive chemotherapy with or without UDCA for 6 months, all followed up for a further 3 months, UDCA treated patients had a trend towards decreased levels of aminotransferases (Saif et al., 2012; Robles-Díaz et al., 2021). In contrast, in an open-label study in 80 pediatric patients with B-cell ALL on maintenance methotrexate treatment, UDCA and vitamin E (antioxidant) treatment showed minimal hepatoprotective benefit (Bordbar et al., 2018).

## Discussion

This review describes the available evidence evaluating the efficacy of hepatoprotective agents in normalizing liver enzyme abnormalities among patients who developed DILI due to anti-cancer therapies. While the mainstay of DILI treatment is the prompt removal of the causative agent, which often leads to spontaneous recovery, this need to be balanced with a need to avoid further deterioration in liver function and understanding of when to initiate appropriate hepatoprotective treatment (Hosack et al., 2023). Covering a range of hepatoprotective drugs including ademetionine, bicyclol, corticosteroids and silymarin, the publications consistently demonstrated improvements in liver enzyme elevations following treatment. However, the number of studies with ani-cancer drugs associated DILI is limited, and often the number of patients involved are small.

The data presented in this review are in line with a growing body of evidence demonstrating the efficacy of hepatoprotective therapies for DILI treatment. While well-designed randomized controlled studies are still needed in many cases to confirm the efficacy of these agents, several real-world database studies have shown efficacy in patients with DILI including with bicyclol, silybin meglumine (water-soluble form of silymarin), and glycyrrhizin injections (Wang et al., 2019; Yao et al., 2022; Zhang et al., 2022; Wang et al., 2021). These studies are important given that DILI is costly, both with regard to healthcare expenditure and in relation to the human toll (Lisi, 2016). DILI during anti-cancer treatment is a particular cause for concern as treatment dose reduction, delay, or discontinuation has the potential to negatively affect clinical outcomes including overall survival (Azad et al., 2018; Mondaca et al., 2020; Regev et al., 2020). Generally, treatment with hepatoprotective agents in the included studies was combined with cessation of causative anti-cancer therapy. However, in some studies hepatoprotective treatments were given prophylactically, enabling patients to continue with their anti-cancer therapy without liver toxicity. For example, in two studies, ademetionine was given from initiation of chemotherapy, reducing the need for course delay and dose reduction (Vincenzi et al., 2012; Vincenzi et al., 2011). In another study, bicyclol was given three times daily on initiation of chemotherapy, significantly lowering the incidence of grade I-IV serum transaminase elevation (Li et al., 2014).

Although ademetionine protects against apoptosis in normal hepatocytes by inhibiting cytochrome C release, it induces apoptosis in liver cancer cell lines HepG2 and HuH-7 (Ansorena et al., 2002). These findings align with the reported chemo-preventive effects of ademetionine demonstrated in an *in vivo* model of chemical hepatocarcinogenesis in rats (Lu et al., 2009). Furthermore, the protective effect of ademetionine supplementation was demonstrated in patients with resected colorectal cancer who were treated with the FOLFOX IV adjuvant regimen (Vincenzi et al., 2011). The available evidence therefore strongly suggests that ademetionine does not compromise the antitumor efficacy of chemotherapy and, instead, may enhance treatment outcomes by protecting normal cells, reducing oxidative stress, and restoring epigenetic balance. Further clinical trials are needed to fully establish the optimal use of ademetionine in combination with chemotherapy and to explore its potential in different cancer types and treatment regimens (Fernández-Ramos et al., 2025).

The clinical manifestations of DILI are heterogeneous and its severity varies from mild liver function elevations to the development of severe liver injury/disease (Hassan and Fontana, 2019). This was highlighted in the current review, which described publications demonstrating a range of liver enzyme elevations necessitating a number of different treatment approaches. The varying clinical approaches taken in these clinical cases highlight the unmet clinical and regulatory challenges and areas for future research that remain in relation to DILI management (Brennan et al., 2022; McGill and Jaeschke, 2019). First, DILI is difficult to predict, diagnose, and treat because its presentation is similar to many hepatobiliary disorders (Ye et al., 2018). As such, there is a need for specific non-invasive diagnostic tests, approved biomarkers and evidence-based diagnostic scales to reduce the reliance on exclusion of other causes of liver disease. The lack of diagnostic tools is compounded by limited mechanistic understanding of toxicity (Grove et al., 2023). Further information in this area would support development and approval of optimal DILI management paradigms. Importantly, this review also highlights a lack of properly designed clinical trials evaluating the efficacy of new treatments as well as older drugs (Andrade et al., 2019; Garcia-Cortes et al., 2020). Further research in this area is required, particularly in the context of anti-cancer therapy-induced DILI, in order to determine the best treatment approaches that will allow patients to continue with their cancer treatment and maximize clinical outcomes.

## Conclusion

This review summarizes the available evidence for the benefits of hepatoprotective agents among patients with DILI due to anti-cancer therapy, with some agents such as ademetionine allowing patients to remain on therapy with a reduced need for dose reductions or delays. However, the review also emphasizes the need for more clinical evidence on the efficacy of hepatoprotective agents in the treatment of DILI caused by anti-cancer therapies, and their potential role as prophylactic therapy. Given the substantial clinical relevance of DILI during anti-cancer treatment, i.e., reduction, delay or discontinuation of anti-cancer therapy and the potential impact on clinical outcomes, these data will provide clinicians with important evidence to support treatment decisions for clinically vulnerable patients.
